# The Proteo‐Transcriptome of Extracellular Vesicles and Particles Is Largely Preserved After Cryopreservation

**DOI:** 10.1002/jex2.70128

**Published:** 2026-05-05

**Authors:** Yohei Nose, Diane Marie del Valle, Tina Ruth Gonsalves, Kevin Tuballes, Ethan Ellis, Hui Xie, Igor Figueiredo, Ruiwei Guo, Avni Chandra, Aana Hahn, Anish Korrapati, Giorgio Ioannou, Rafael Cabal, Swapnil Tichkule, John F. Fullard, Panos Roussos, Pedro Silva, Angelo Amabile, Jarod Morgenroth‐Rebin, Travis Dawson, Raphael Merand, Kai Nie, Zhihong Chen, Sharon Nirenberg, Brian Brown, Seunghee Kim‐Schulze, Andrew Kaufman, Raja Flores, Laura Zuluaga, Kristin Beaumont, Robert Sebra, Natasha Kyprianou, Kyrollis Attalla, Ketan Badani, Ash Tewari, Navneet Dogra, Sacha Gnjatic, Edgar Gonzalez‐Kozlova

**Affiliations:** ^1^ Department of Immunology and Immunotherapy Icahn School of Medicine at Mount Sinai New York New York USA; ^2^ Genetics and Genomic Sciences Icahn School of Medicine at Mount Sinai New York New York USA; ^3^ Human Immune Monitoring Center Icahn School of Medicine at Mount Sinai New York New York USA; ^4^ Department of Pathology Icahn School of Medicine at Mount Sinai New York New York USA; ^5^ Department of Psychiatry Icahn School of Medicine at Mount Sinai New York New York USA; ^6^ Center for Disease Neurogenomics Icahn School of Medicine at Mount Sinai New York New York USA; ^7^ Center for Precision Medicine and Translational Therapeutics James J. Peters VA Medical Center Bronx New York USA; ^8^ Department of Thoracic Surgery Icahn School of Medicine at Mount Sinai New York New York USA; ^9^ Department of Urology Icahn School of Medicine at Mount Sinai New York New York USA

**Keywords:** extracellular vesicles, fresh and frozen, olink, proteomics, RNA‐seq, storage condition, tissue sample

## Abstract

Extracellular vesicles and particles (EVPs) are small lipid‐bilayer membrane structures containing proteins, lipids, and nucleic acids that play crucial roles in tumorigenesis, metastasis, and immunomodulation. The extent to which frozen tissues faithfully reflect the biology of fresh tissues remains unclear. To address this issue in the context of EVPs, we analysed proteo‐transcriptomic differences in EVP cargo between fresh and frozen human tissues. First, we extracted EVPs from tumour and adjacent normal tissues from cancer patients with a combination of ultrafiltration and size‐exclusion chromatography. Next, we profiled the protein and RNA contents using spectroscopy methods. Further, we used Olink and mixed‐effect models to investigate the differences between fresh and frozen protein contents. Our results show that cryopreservation has little effect on protein concentration, particle size, and proteo‐transcriptomics of EVPs. These findings support the feasibility of using frozen specimens for EVPs research, potentially expanding access to large biobanks that house frozen specimens while mitigating reliance on fresh tissue samples, and in doing so, overcoming a major obstacle in human research.

## 1 Introduction

Extracellular vesicles and particles (EVPs) are membrane‐derived vesicles released by cells into the extracellular space, playing key roles in intercellular communication and the regulation of various biological processes (Cheng and Hill 2022; Kalluri and McAndrews 2023). EVPs are commonly found in biological fluids and encapsulate a diverse cargo of lipids, metabolites, nucleic acids, and proteins that reflect their cell of origin (Chen et al. 2022; Dogra et al. 2024; Miceli et al. 2024; Van Niel et al. 2018). In contrast, EVPs isolated directly from tissue samples offer unique advantages, including a more enriched and accurate representation of tissue‐specific features and the local microenvironment (Li et al. 2021; Zhi et al. 2022). While extensive research has been conducted on EVPs derived from body fluids or cell culture systems, there remains a relative scarcity of studies focused on the isolation and characterisation of tissue‐derived EVPs (Al Hrout et al. 2023; Crescitelli et al. 2021; Hurwitz et al. 2019; Li et al. 2021; Lin et al. 2024; Morgan et al. 2024; Zhi et al. 2022; Zhu et al. 2025).

Obtaining fresh tissue and minimising ischaemia time of clinical samples remains a major challenge in cancer research. For instance, successful single‐cell RNA sequencing (scRNA‐seq) typically requires rapid tissue dissociation, which is often not feasible immediately after sample collection, necessitating freezing for later processing. Consequently, the impact of sample preservation on downstream RNA‐seq analysis has been actively investigated (Slyper et al. 2020; Wang et al. 2023). A similar concern arises in the context of EVP analysis, as the biological properties and molecular cargo of EVPs may be influenced by storage conditions and EVP purification procedures. Müller et al. reported that freezing and thawing of plasma reduced exosome purity without affecting biological activity (Muller et al. 2014), and Gelibter et al. observed a time‐dependent decrease in extracellular vesicle concentration and sample purity following frozen storage of EVPs collected from tissue, blood, and cell cultures (Gelibter et al. 2022). In contrast, Tsamchoe et al. found no significant differences in protein or mRNA profiles between fresh and frozen plasma samples (Tsamchoe et al. 2023). Although several studies have explored the effects of storage on EVPs derived from biofluids, limited data are available regarding tissue‐derived EVPs.

Most reported methods for isolating tissue‐derived EVPs rely on fresh tissue, and to the best of our knowledge, only a few studies have explored the extraction of EVPs from either fresh or frozen tissue samples (Al Hrout et al. 2023; Hurwitz et al. 2019; Yang et al. 2024). Moreover, no studies to date have directly compared the cargo characteristics of EVPs isolated from fresh versus frozen tissues in downstream applications. As research in the EVP field continues to expand, it is essential to understand how sample storage conditions influence EVP cargo composition. The use of frozen tissue samples enables simultaneous or batched analysis of specimens from different patients or sources, offering practical advantages in clinical and translational research (Witwer et al. 2013). Therefore, it is critical to investigate the potential effects of freezing and thawing on EVP composition and content.

In this study, we investigated the overall protein (including an immune‐oncology Olink protein panel) and RNA profiles of fresh and frozen tissue‐derived EVPs. Our results indicate that, except for a few markers, freezing did not significantly impact the proteomic profiles of tissue‐derived EVPs. These findings support the feasibility of using frozen tissue samples for EVP analysis, broadening the potential of archived biobank specimens in future EVP research.

## 2 Methods

### 2.1 Specimen Collection

This study was reviewed and approved by the Icahn School of Medicine at Mount Sinai Program for the Protection of Human Subject office (STUDY 21–01308). Patients with a confirmed cancer diagnosis undergoing surgical resection of the tumour were approached for study participation. Inclusion criteria were broad and included being ≥ 18 years of age, having a cancer diagnosis at the time of consent, and having a resectable tumour for surgery of ≥ 2 cm. Exclusion criteria included patients participating in clinical trials or other research studies requiring specimen collection. Patients were referred to the research study by their surgeon or treating oncologist. All consents were obtained, and the appropriate institutional documentation has been archived.

The surgical procedure was conducted as per standard clinical practice. Resected tissue specimens were processed by the Mount Sinai Health System Department of Pathology. Once diagnostic pathology was completed, any residual tumour and/or normal adjacent tissue were processed for research purposes. All samples were identified. Tumour and adjacent normal‐adjacent tissue samples were collected from patients diagnosed with lung (*n* = 5), prostate (*n* = 1), and kidney (*n* = 3) cancers. Serum samples from peripheral blood specimens for the Olink proteomic assay were obtained from two lung cancer patients and two healthy donors, while PBMC samples were collected from two cancer patients and four healthy donors. These individuals are different from the patients providing tissue samples.

### 2.2 Tissue Sample Collection and Digestion

Each tissue sample was divided into one fresh sample and two frozen samples. The frozen samples were thawed using two different protocols: frozen‐fast (Fast) or frozen‐slow (Slow). Fresh tissues were processed immediately after collection for separation of cells and soluble remains or supernatants using gentleMACS standard protocols described below. In contrast, Fast tissues were snap‐frozen post‐collection and subsequently thawed rapidly, whereas Slow tissues underwent a gradual thawing process. During tissue digestion, the tissue supernatant was collected after centrifugation and stored at ‐80°C. Collected tissues were weighed and subdivided into approximately equal portions across the protocols. Digestion buffers were prepared proportionally to tissue weight using Roswell Park Memorial Institute (RPMI) 1640 medium and a defined enzyme mix and processed in gentleMACS C Tubes. For tissues ≤0.2 g, 2.2 mL RPMI 1640, 100 µL Enzyme H, 50 µL Enzyme R, and 12.5 µL Enzyme A were used; for tissues >0.2 g, the volumes were 4.7 mL RPMI 1640, 200 µL Enzyme H, 100 µL Enzyme R, and 25 µL Enzyme A. Tissue fragments were transferred into the enzyme‐containing gentleMACS C Tubes. After a brief centrifugation to collect material at the tube bottom, the tissue was resuspended and filtered through a 70 µm cell strainer into a 50 mL conical tube. The cells were washed with 20 mL RPMI 1640, followed by centrifugation at 300 × g for 7 minutes. The resulting supernatant was collected for downstream analysis.

### 2.3 Freezing Protocol (Frozen) for Tissue Samples

A cryopreservation solution consisting of 10% dimethyl sulfoxide and 90% foetal bovine serum was prepared in 10 mL volumes using 50 mL conical tubes. One millilitre of this solution was dispensed into each cryovial designated for tissue freezing and pre‐chilled on ice. Dissected tissue samples were then added to the cryovials, which were immediately placed in a controlled‐rate freezing container and stored at −80°C, allowing for a cooling rate of approximately 1°C per minute. On the following day, the cryovials were transferred to liquid nitrogen for long‐term storage.

### 2.4 Thawing (Fast and Slow) Protocol for Tissue Samples

Cryovials were removed from −80°C storage and immediately immersed in a 37°C water bath. Once a small amount of ice remained, the vials were withdrawn from the bath to avoid overheating of the tissue. For the Fast protocol, the entire vial contents (∼1 mL) were transferred into a 15 mL conical tube containing 10 mL of pre‐warmed RPMI 1640 medium. After the tissue settled at the bottom, the supernatant medium was completely aspirated. For the Slow protocol, 1 mL of pre‐warmed complete RPMI 1640 was slowly added dropwise into the cryovial to initiate gentle thawing. The contents of the vial (∼2 mL total) were then transferred to a 15 mL conical tube, and additional medium was gradually added in a stepwise manner until the final volume reached approximately 8 mL. The tissue was allowed to settle, and the supernatant medium was aspirated.

### 2.5 Sample Pre‐Processing

Thawed tissue supernatants or serum samples were first centrifuged at 300 × g for 10 min, followed by 2000 × g for 20 min at 4°C to remove cellular debris and aggregates. The resulting supernatant was then subjected to the ultrafiltration (UF) step, as described below. PBMCs were isolated by Ficoll‐Paque density gradient centrifugation. Isolated PBMCs were resuspended at a density of 2 × 10^6^ cells/mL in serum‐free medium (X‐VIVO 15; Lonza) and cultured for 72 h. After incubation, culture supernatants were collected and centrifuged under the same conditions (300 × g for 10 min, followed by 2000 × g for 20 min at 4°C) to remove residual cells and debris.

### 2.6 EVP Isolation

#### 2.6.1 Ultrafiltration (UF)

After pre‐processing, each tissue‐supernatant sample was concentrated using a pre‐rinsed, sterilised centrifugal filter unit with a molecular weight cut‐off (MWCO) of 100 kDa, either Sartorius Vivaspin (VS2061) or Centricon Plus‐70 (Millipore), by centrifugation at 3500 × g for 30 min at 4°C (Tkach et al. 2022). This UF step was repeated as needed until the final volume was reduced to less than 500 µL. Protein and RNA concentrations were measured before and after concentration using a NanoDrop spectrophotometer (Thermo Fisher Scientific, Wilmington, DE, USA).

#### 2.6.2 Size‐Exclusion Chromatography (SEC)

After concentration to a final volume of less than 500 µL, samples were loaded onto PBS‐rinsed 70 nm qEV SEC columns (Izon Science, Cambridge, MA, USA) for size separation. A total of twenty 1250 µL fractions were collected sequentially, following the manufacturer's protocol (Navajas et al. 2019; Veerman et al. 2021). The absorbance of each fraction at 260, 280, and 498 nm was measured using a NanoDrop to estimate RNA and protein concentrations. All fractions were subsequently aliquoted and stored at –80°C until further analysis.

### 2.7 EVP Characterization

#### 2.7.1 Western Blotting

SEC‐separated EVP samples (fractions 1–4 and 5–10) were pooled separately and further concentrated using 10 kDa molecular weight cutoff filters (Amicon Ultra‐4, Millipore). Protein concentrations were measured using a NanoDrop, and the amount of protein loaded per lane was adjusted to 5–30 µg. Samples were diluted in 4× loading buffer and resolved by SDS‐PAGE on precast polyacrylamide gels (NuPAGE Bis‐Tris Gel, Invitrogen). Proteins were transferred onto nitrocellulose membranes using the iBlot 2 Transfer Stacks (Invitrogen) and blocked with 5% milk in Tris‐Buffered Saline with Tween 20 (TBST) for 1 hour at room temperature. Membranes were then incubated overnight at 4°C with primary antibodies specific to the target proteins. The following day, membranes were washed three times in TBST and incubated with a goat anti‐rabbit HRP‐conjugated secondary antibody. Chemiluminescent detection was performed using ECL Prime Western Blotting Detection Reagent. The following primary antibodies were used: rabbit anti‐human calnexin (clone EPR3633(2), Abcam), rabbit anti‐human CD9 (clone EPR23105‐125, Abcam), and rabbit anti‐human TSG101 (clone EPR7130(B), Abcam). According to the Minimal Information for Studies of Extracellular Vesicles (MISEV) guidelines (Welsh et al. 2024), CD9 (category 1) and TSG101 (category 2) are considered hallmark EV markers, while calnexin (category 4) serves as a negative or exclusion marker, being associated with intracellular compartments.

#### 2.7.2 Dynamic Light Scattering (DLS)

Dynamic light scattering (DLS) was performed to assess the particle size distribution of fractions separated by SEC. DLS measures the Brownian motion of particles in suspension by detecting temporal fluctuations in scattered light intensity from a monochromatic laser beam, allowing calculation of hydrodynamic particle diameters (Khan et al. 2022; Lim et al. 2013; Szatanek et al. 2017). In the comparison of pooled fractions shown in Figure 2D,E SEC fractions were grouped into two pools (fractions 1–4 and 5–10), followed by further concentration using centrifugal filters with a 10 kDa molecular weight cut‐off. Protein concentrations were measured and adjusted to 0.1–1 mg/mL with PBS. Samples were diluted 1:250 in PBS before measurement and loaded into folded capillary cells, which were pre‐rinsed with filtered ultrapure water. DLS measurements were performed using a Zetasizer Pro (Malvern Instruments, Malvern, UK) (Gečys et al. 2022), with each sample analysed in triplicate. The mean particle size from the three measurements was used for downstream analysis (Tan et al. 2021).

#### 2.7.3 RNA Extraction and Capillary Electrophoresis of RNA

MicroRNA (miRNA) and total RNA were extracted from EVPs using the miRNeasy Micro Kit (QIAGEN, Cat# 217084), according to the manufacturer's protocol. Briefly, the lysis reagent was added to each sample for disruption and homogenisation. Chloroform was then added, mixed vigorously, and incubated at room temperature for 3 min. Samples were centrifuged at 12,000 × g for 15 min at 4°C, and the upper aqueous phase was carefully transferred to a new collection tube without disturbing the lower phase. Next, 1.5 volumes of 100% ethanol were added, mixed, and applied to the spin column. The column‐bound RNA was washed three times with the provided wash solutions. 500 µL of 80% ethanol was added and centrifuged at 8000 × g for 2 min. The spin column was then transferred to a new collection tube and centrifuged again at 8000 × g for 5 min to fully dry the membrane. Finally, RNA was eluted in 14 µL of RNase‐free water by centrifugation at 8000 × g for 1 min. Eluted RNA was stored at −80°C until analysis. RNA quality and integrity were assessed using the Agilent 2100 Bioanalyzer with the RNA 6000 Pico Kit (Agilent Technologies), and high‐sensitivity RNA reagents were used for all EVP samples. RNA concentration, length distribution, and RNA Integrity Number equivalent (RINe) were calculated using Agilent software. RNA purity was further evaluated before and after extraction by measuring the 260/280 absorbance ratio using a NanoDrop.

#### 2.7.4 Transcriptomics of Tissue‐Derived EVPs

RNA quality was assessed by bioanalyzer (Agilent 2100 Bioanalyzer, RNA 6000 Pico Kit, Agilent Technologies). cDNA libraries were prepared for small RNAs using the SMARTer smRNA‐seq Kit for Illumina (Takara Bio 635030). A total of 18 cycles of PCR were carried out to obtain a good yield of cDNA from tissue, cells, and EVs. Final library quality was verified with Qbit and Bioanalyzer. Negative (no RNA) and positive controls provided expected results. Next‐generation RNA‐seq was performed using a HiSeq 4000 (Illumina), 100 base pair, single‐end reads.

#### 2.7.5 Genome Mapping

For quantification of gene expression, raw reads were aligned to the latest Ensembl GRCh38.p13 (GCA_000001405.28) using the bowtie aligner (version 2.5.4b). FeatureCounts were then used to map the aligned reads to the GENCODE v26 primary gene annotation, including transcripts corresponding to ncRNAs such as lncRNA, miRNA, as well as protein‐coding RNA. To maximisze recovery and minimise the noise, multimapping reads were quantified up to *m* = 10 and distributed using a unique reads mapping distribution, as described in the most recent best practices protocols.

### 2.8 Pathway Analysis

To effectively compare, not only enriched genes, but also against enriched proteins, pathway enrichment analyses are performed using the enrichR package in R. Specifically, we referenced databases including Kyoto Encyclopedia of Genes and Genomes (Versions: 2013, 2015, 2016, 2019, 2021), Gene Ontology Molecular Function (Versions: 2013, 2015, 2017, 2017b, 2018, 2021), Gene Ontology Cellular Component (Versions: 2013, 2015, 2017, 2017b, 2018, 2021), Gene Ontology Biological Process (Versions: 2013, 2015, 2017, 2017b, 2018, 2021), Reactome (Version: 2016), and WikiPathways (Version: 2019). The top 1500 enriched genes and all of the proteomic signatures were used for the pathway analysis. Top 10 genomic and proteomic pathways from each database with FDR below 0.05 and at least three enriched genes present were selected.

#### 2.8.1 Soluble Factor and Surface Protein Profiling by Multiplex Proximity Extension Assay

The EVP proteins were analysed using the Olink multiplex assay platform with Inflammation and Immuno‐oncology panels (Olink Bioscience, Uppsala, Sweden), according to the manufacturer's instructions (Hjelm et al. 2011). The Olink assay is based on proximity extension assay (PEA) technology, in which pairs of matched antibodies are conjugated with unique DNA oligonucleotides. Upon simultaneous binding to the same target protein, the oligonucleotides are brought into close proximity, enabling DNA polymerase to extend and create a unique DNA sequence. This sequence is then quantified by real‐time PCR or Next‐Generation Sequencing (NGS), providing highly specific and sensitive protein detection. Each panel quantifies 92 target proteins, including cytokines, chemokines, antigens, and soluble immune checkpoint molecules (Monjazeb et al. 2021; Parra et al. 2024). EV and EP fractions were extracted and analysed from five tumour tissues (three lung, one prostate, and one kidney cancer) under each storage protocol. In addition, serum or plasma samples were analysed in three groups: original (pre‐SEC), EVs, and EPs. Moreover, PBMCs were cultured and their supernatants were similarly separated into original, EVs, and EPs. Protein and RNA concentrations were measured and adjusted with PBS to meet the assay requirements. Control samples included serum from a healthy donor (HD1017) and medium samples (X‐VIVO, RPMI 1640, and PBS), which were processed and analysed in parallel.

#### 2.8.2 Olink Data Analysis

Protein levels were normalised using internal positive and negative controls and quantified as log_2_‐scale Normalised Protein expression (NPX) values, which were subsequently used for downstream analyses. Data distribution for each sample was assessed, and samples flagged with warnings after NPX conversion were carefully inspected (Galsky et al. 2023). To account for multiple comparisons, the FDR was calculated.

### 2.9 Statistical Analysis

Statistical analyses were performed using the Student's *t*‐test or Mann–Whitney U test for comparisons between two independent groups, as appropriate. Paired samples were analysed using the nonparametric Wilcoxon matched‐pairs signed‐rank test. Comparisons of categorical and continuous variables were conducted as described in the corresponding figure legends. A value of *P* < 0.05 was considered to be significant. All statistical analyses were performed using R, R‐Studio, and JMP Pro 17 Discovery (SAS Institute Inc., Cary, NC). The code used for analysis is available on GitHub at https://github.com/eegk/FreshFrozenEVPs.

**Quality controls**: The analysis for all datasets (Olink, RNA‐seq) was performed in R software using a mixed linear model strategy to adjust for relevant clinical and technical variables. The data distributions for markers and cell populations for all assays were investigated as part of a routine quality control to identify biases, and corrected as follows: (1) samples with more than 50% missing values in any analyte were excluded; (2) Olink analytes that were under the limit of detection in more than 50% of samples were excluded. (3) QC analyses were used to identify biases such as low detection and poor‐quality samples. For Olink, healthy positive controls were employed, and the medium was used as a negative control. For RNA‐seq, standardised HHRR was used as a positive control, and non‐human microRNA 273 was used as a negative control to avoid sequencing artifacts.
**Variance analysis**: Sample variance profiles were performed to assess the effect of covariates with assay data (Olink, RNA‐seq) using the package variancePartition/Dream on R. Covariates with less than 5% effect on the model were excluded from modelling.
**Adjust *p*‐values for multiple comparisons**: For multi‐omic assays (Olink, RNA‐seq), We applied moderate *T*‐test statistics. We adjusted *p*‐values using the Benjamini and Hochberg method (1995). This helps to control the false discovery rate, the expected proportion of false discoveries amongst the rejected hypotheses. Nonetheless, throughout the manuscript we show nominally significant results as *p* < 0.05 and adjusted *p* values represented as FDR < 0.05.
**Differential expression**: Differential protein and gene expression analysis was performed in R using the packages Dream, lme4, from bioconductor. The mixed effect models were built using the relevant covariates. For Olink, the independent variables were individual protein levels (NPX). The results were visualised using pheatmap and ggplot2 packages.
**Correlation analyses**: We used cor, corrplot, pvclust, and Hmisc packages in R‐Stats to perform Pearson (linear) and Spearman (non‐linear) correlations between analytes and relevant variables.

## 3 Results

### 3.1 Protein and RNA Were Enriched by Ultrafiltration, and Bimodal Peaks Were Confirmed After Size‐Exclusion Chromatography

**Figure 1:**
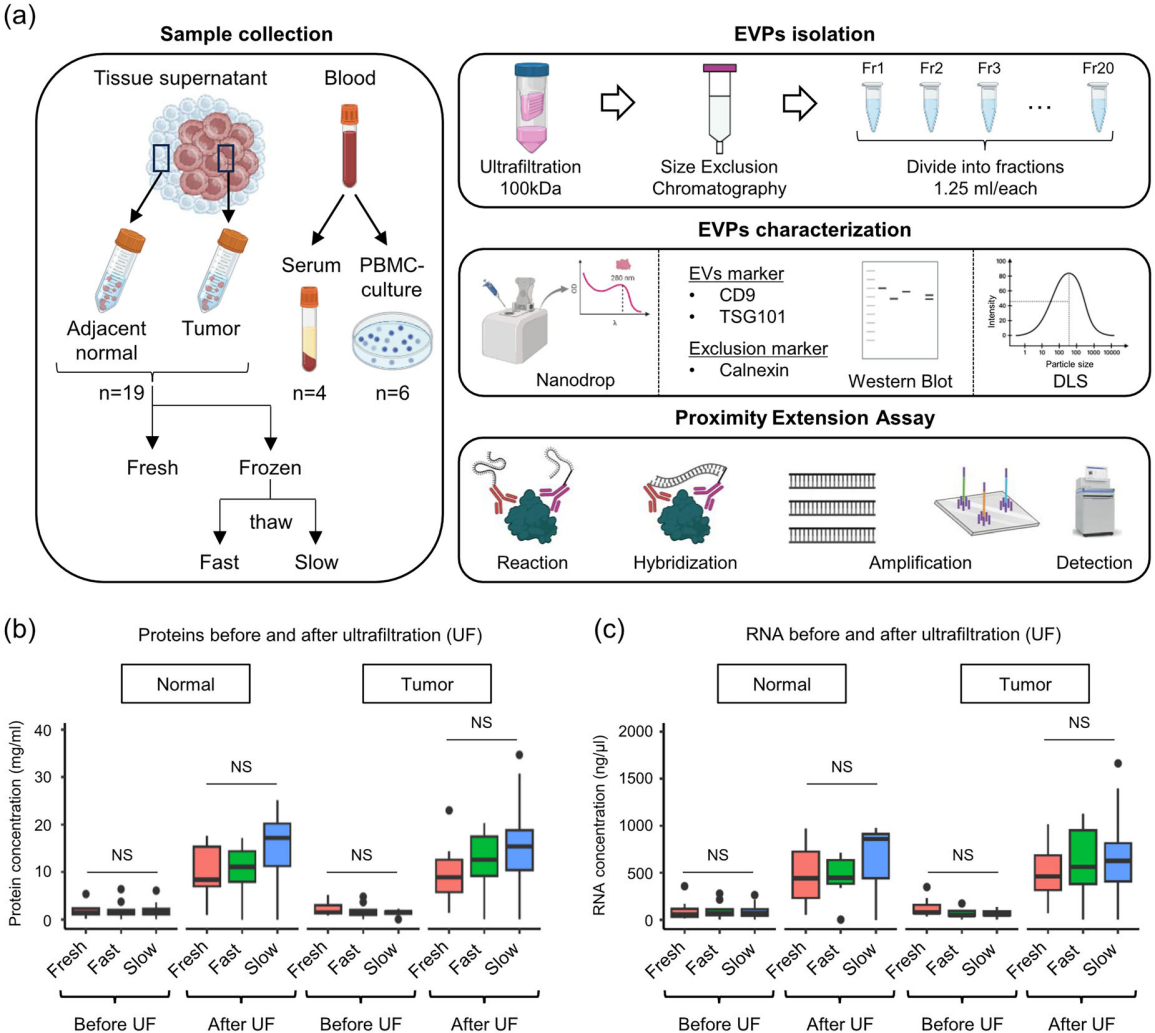
**Workflow and quality control**. (A) Overview of the study and the workflow of sample collection (tissue: *n* = 19, serum: *n* = 4, PBMC: *n* = 6) and experimental design. (B–C) Protein (B) and RNA (C) concentration before and after ultrafiltration (UF) for tumour and normal tissue samples under the three storage protocols (Fresh, Fast, and Slow). (B–C) Values are indicated as medians. The significance of differences was calculated using the nonparametric Wilcoxon matched‐pairs signed‐rank test (tumour = 11, normal = 8). “NS” indicates no statistically significant difference.

To broadly compare EVP composition in fresh and frozen tissues, we used surgical resections from patients with lung (non‐small‐cell), prostate, or kidney cancer (clear cell). In total, 11 tumour and 8 adjacent normal tissue samples were collected (Figure 1A, Table 1 and Table S1) from 9 patients. We used the Standard PREanalytical Code (SPREC) that is a globally recognized best practice for documenting and controlling pre‐assay specimen handling to record specimen handling and freezing times. This code is promoted by ISBER, it strengthens the foundation of biomarker studies by making preanalytical metadata systematic, comparable, and transparent (Betsou et al. 2025; Lehmann et al. 2012; López‐Guerrero et al. 2023; Skoworonska et al. 2023).

**Table 1:** A list of tissue weights and cell counts for all 19 samples.

Sample	Patient	Tissue type	Cancer type	Storage	Weights (g)	Number of cells (M)
1	7002	tumour	lung	fresh	0.1000	1.2750
1	7002	tumour	lung	frozen‐fast	0.0740	0.1060
1	7002	tumour	lung	frozen‐slow	0.0740	0.3400
2	7002	normal	lung	fresh	0.1530	0.1180
2	7002	normal	lung	frozen‐fast	0.0895	0.0006
2	7002	normal	lung	frozen‐slow	0.0895	0.0005
3	7003	tumour	lung	fresh	0.2270	3.5600
3	7003	tumour	lung	frozen‐fast	0.0705	N/A
3	7003	tumour	lung	frozen‐slow	0.0705	0.0145
4	7003	normal	lung	fresh	0.3590	0.6340
4	7003	normal	lung	frozen‐fast	0.0920	N/A
4	7003	normal	lung	frozen‐slow	0.0920	0.0025
5	7009	tumour	lung	fresh	0.0530	0.6360
5	7009	tumour	lung	frozen‐fast	0.0290	0.2950
5	7009	tumour	lung	frozen‐slow	0.0290	0.1020
6	7009	normal	lung	fresh	0.0880	0.0052
6	7009	normal	lung	frozen‐fast	0.0615	0.0060
6	7009	normal	lung	frozen‐slow	0.0615	0.0090
7	7010	tumour	lung	fresh	0.1030	0.4400
7	7010	tumour	lung	frozen‐fast	0.0885	0.6500
7	7010	tumour	lung	frozen‐slow	0.0885	0.8000
8	7010	normal	lung	fresh	0.2090	1.2200
8	7010	normal	lung	frozen‐fast	0.2085	0.6000
8	7010	normal	lung	frozen‐slow	0.2085	0.6300
9	7020	tumour	lung	fresh	0.0990	0.2830
9	7020	tumour	lung	frozen‐fast	0.0495	0.0190
9	7020	tumour	lung	frozen‐slow	0.0495	0.0250
10	7020	normal	lung	fresh	0.0790	0.5320
10	7020	normal	lung	frozen‐fast	0.0395	0.0200
10	7020	normal	lung	frozen‐slow	0.0395	0.0350
11	7015	tumour‐core	prostate	fresh	0.0800	0.0052
11	7015	tumour‐core	prostate	frozen‐fast	0.0580	0.0063
11	7015	tumour‐core	prostate	frozen‐slow	0.0580	0.0113
12	7015	tumour‐peri	prostate	fresh	0.0850	0.0420
12	7015	tumour‐peri	prostate	frozen‐fast	0.0880	0.5950
12	7015	tumour‐peri	prostate	frozen‐slow	0.0880	0.0207
13	7008	tumour‐core	kidney	fresh	0.1700	1.6500
13	7008	tumour‐core	kidney	frozen‐fast	0.1750	0.3544
13	7008	tumour‐core	kidney	frozen‐slow	0.1750	0.5600
14	7008	tumour‐peri	kidney	fresh	0.1200	1.8500
14	7008	tumour‐peri	kidney	frozen‐fast	0.1200	0.3092
14	7008	tumour‐peri	kidney	frozen‐slow	0.1200	0.3088
15	7008	normal	kidney	fresh	0.0600	1.1800
15	7008	normal	kidney	frozen‐fast	0.0650	0.0103
15	7008	normal	kidney	frozen‐slow	0.0650	0.0058
16	7018	tumour‐peri	kidney	fresh	0.1250	1.1000
16	7018	tumour‐peri	kidney	frozen‐fast	0.1175	0.0100
16	7018	tumour‐peri	kidney	frozen‐slow	0.1175	0.0600
17	7018	normal	kidney	fresh	0.0700	0.5320
17	7018	normal	kidney	frozen‐fast	0.0980	0.0280
17	7018	normal	kidney	frozen‐slow	0.0980	0.0000
18	7006	tumour‐core	kidney	fresh	0.2700	1.3100
18	7006	tumour‐core	kidney	frozen‐fast	0.2700	2.5230
18	7006	tumour‐core	kidney	frozen‐slow	0.2700	0.4770
19	7006	normal	kidney	fresh	0.2500	0.7120
19	7006	normal	kidney	frozen‐fast	0.2500	0.0786
19	7006	normal	kidney	frozen‐slow	0.2500	0.0720

*Note*: An abbreviation is used for the following: Tumour‐peri = tumour periphery; and tumour‐core = tumour center

The 19 samples were divided into two storage protocols: fresh and frozen. Additionally, as a secondary endpoint, separate aliquots of frozen specimens were subjected to two thawing methods in parallel: fast thaw and slow thaw, referred to thereafter as frozen‐fast (Fast) and frozen‐slow (Slow), respectively (Methods). Tissue weight and cell number were mildly correlated (Pearson = 0.5, *P* < 0.05, Figure S1A). There were no significant differences (*P* > 0.05) in tissue weight across the protocols (Figure S1B). Also, no significant differences (*P* > 0.05) were observed in protein or RNA concentrations, either before or after ultrafiltration (UF), among the Fresh, Fast, and Slow samples, in both tumour and normal tissues (Figure 1B,C).

**Figure 2:**
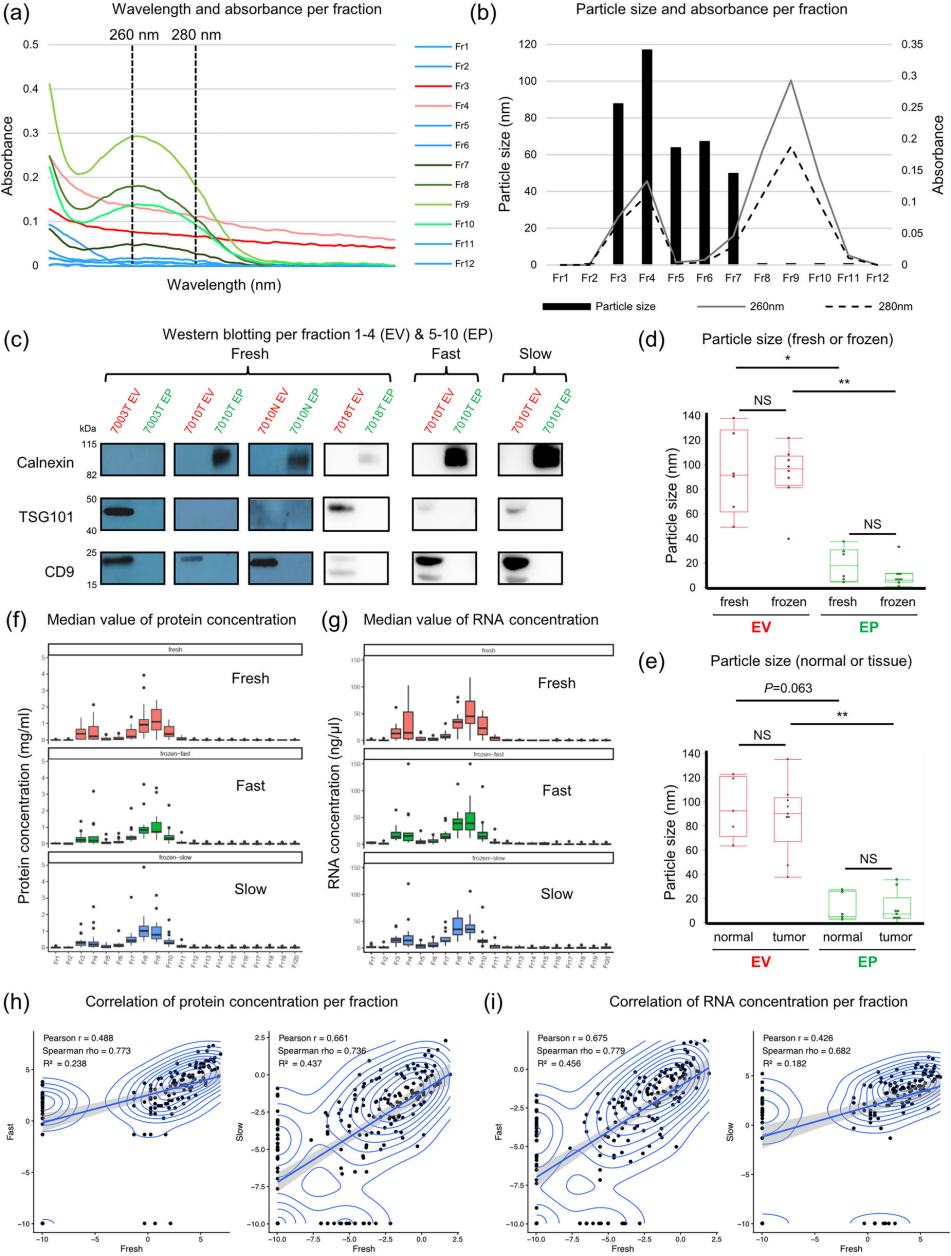
**Isolation and characterization of EVP**. (A) A representative example (7006 tumour fresh) of wavelength and absorbance of fraction 1–12, divided by size‐exclusion chromatography. Vertical dotted lines are shown at 260 nm and 280 nm. (B) A representative graph of the combination of particle size measured by DLS and absorbance (260 and 280 nm) by Nanodrop in a tissue sample (7006 tumour fresh). (A–B) Fractions from 13 onward were omitted due to negligible absorbance. (C) Western blotting analysis of calnexin, TSG101, and CD9 in fraction 1–4 (EV) and 5–10 (EP). TSG101 and CD9 were used as extracellular vesicles (EVs) markers, and calnexin was used as EVs exclusion marker. (D) Particle size in each fraction among fresh and frozen samples (fresh: *n* = 6, frozen: *n* = 8). (E) Particle size in each fraction among tumour and normal samples (tumour: *n* = 9, normal: *n* = 5). (D–E) Values are indicated as medians. The significance of differences between fraction 1–4 (EV) and 5–10 (EP) in the same sample was calculated using the nonparametric Wilcoxon matched‐pairs signed‐rank test. The significance of differences between fresh and frozen or tumour and normal samples was calculated using the nonparametric Mann–Whitney U test (*P < 0.05, **P < 0.01, ***P < 0.001). “NS” indicates no statistically significant difference. (F–G) Median value of protein (F) and RNA (G) concentrations per fraction for Fresh, Fast, and Slow (F: tumour: *n* = 10, normal: *n* = 7, G: tumour: *n* = 8, normal: *n* = 5). (H–I) Correlation of protein (H) and RNA (I) concentrations for each fraction between fresh and frozen samples. Left, fresh versus frozen‐fast; right, fresh versus frozen‐slow. The Spearman's rank correlation coefficient (*ρ*) was calculated.

Next, we isolated EVPs by size‐exclusion chromatography (SEC) and collected fractions of 1.25 mL each (Figure 1A). Following SEC, we recorded the absorbance of each fraction at 260 nm (nucleic acids) and 280 nm (protein) (Figure 2A). Two peaks were recorded, the first in fractions (F) 3–4 and F8‐10 for both 260 and 280 nm. The other fractions showed extremely low or undetectable absorbance. Furthermore, protein and RNA concentrations across the SEC‐separated fractions showed bimodal distribution patterns in all samples, with prominent peaks in F3‐4 and F8‐10, respectively (Figure S1C,D).

To investigate whether UF before SEC affects the absorbance per fraction, a tissue (7005 Normal, fresh) was processed under three conditions: (1) without UF, (2) with 10 kDa UF, and (3) 100 kDa UF. Each sample was subsequently separated by SEC, and absorbance was measured (Figure S2A). The second peak (F8‐10) in the conditions without UF and 10 kDa UF conditions appeared slightly later compared to 100 kDa UF, suggesting the presence of smaller molecules in those fractions. In contrast, the first peak (F3‐4) remained consistent across all conditions. Furthermore, in the serum and peripheral blood mononuclear cell (PBMC)‐culture samples analysed for Olink proteomics, the first peak was less prominent than the second when compared to the tissue samples (Figure S2B,C). These findings suggest that different tissue sources may contain different proportions of EVP subtypes, and that isolation of EVPs (with or without UF) remains consistent within a tissue type.

### 3.2 Molecular Characterisation Using Dynamic Light Scattering and Western Blotting Distinguishes Extracellular Vesicles (EVs) From Extracellular Particles (EPs)

Each SEC‐separated fraction and the pooled fractions (F1‐4 and F5‐10) were concentrated by UF and analysed by dynamic light scattering (DLS) (Figure 1A). A representative result from a tumour sample (7006 fresh, most similar to the average of all samples), including the particle size distribution and its integration with absorbance data, is shown (Figure 2B). As expected, particle size was the largest in F3‐4 and gradually decreased from F4 to 12. Interestingly, F5‐6 were found to contain particles with diameters around 50 nm, while showing minimal absorbance at 260 and 280 nm, indicating a negligible presence of proteins and nucleic acids and/or a low number of vesicles. The first absorbance peak (F3‐4) contained a high abundance of particles approximately 100 nm in diameter, whereas the second peak (F8‐10) comprised smaller particles less than 50 nm in diameter.

Additionally, western blotting was performed on 18 samples (Figure 1A and Table 2). As recommended by the MiSEV2025 guidelines, extracellular vesicle (EVs) markers CD9 and TSG101 were primarily found in F1‐4 (CD9 in 7/9 samples, TSG101 in 5/9), while the non‐EV marker calnexin appeared mostly in F5‐10 (5/9 samples). These results indicate that fractions 1–4 are enriched in EVs, whereas F5‐10 contain little to no EVs (Figure 2C). Together, these findings indicate that the first absorbance peak (F1‐4) represents EVs around 100 nm, while the second peak (F5‐10) represents small extracellular particles (EPs), ≤ 50 nm in size (Welsh et al. 2024).

**Table 2:** A list of samples for western blotting.

Patient	Tissue type	Cancer type	Storage	Fraction	Calnexin	TSG101	CD9
7003	tumour	lung	fresh	1–4	not detected	detected	detected
7003	tumour	lung	fresh	5–10	not detected	not detected	not detected
7003	normal	lung	fresh	1–4	not detected	not detected	not detected
7003	normal	lung	fresh	5–10	not detected	not detected	not detected
7009	tumour	lung	fresh	1–4	not detected	not detected	detected
7009	tumour	lung	fresh	5–10	not detected	not detected	not detected
7010	tumour	lung	fresh	1–4	not detected	not detected	detected
7010	tumour	lung	fresh	5–10	detected	not detected	not detected
7010	normal	lung	fresh	1–4	not detected	not detected	detected
7010	normal	lung	fresh	5–10	detected	not detected	not detected
7010	tumour	lung	frozen‐fast	1–4	not detected	detected	detected
7010	tumour	lung	frozen‐fast	5–10	detected	not detected	not detected
7010	tumour	lung	frozen‐slow	1–4	not detected	detected	detected
7010	tumour	lung	frozen‐slow	5–10	detected	not detected	not detected
7018	tumour	kidney	fresh	1–4	not detected	detected	detected
7018	tumour	kidney	fresh	5–10	detected	not detected	not detected
7018	normal	kidney	frozen	1–4	not detected	detected	not detected
7018	normal	kidney	frozen	5–10	not detected	not detected	not detected

### 3.3 Comparison of EVs and EPs Protein or RNA Concentration Between Fresh and Frozen Samples

Particle sizes of pooled F1‐4 (EV) and 5–10 (EP) were measured and compared between fresh and frozen protocols (Figure 2D). No significant (*P* > 0.05) difference in particle size was observed between fresh and frozen samples in either EV or EP fractions. Furthermore, no significant (*P* > 0.05) difference in particle size was observed between tumour and adjacent normal tissues within the same fraction groups (Figure 2E).

Subsequently, the protein and RNA concentrations of each SEC‐separated fraction were compared for each storage protocol (Figure 2F,G). The correlation of protein and RNA concentrations between fresh and either fast or slow freezing protocols was relatively high, suggesting that storage conditions do not significantly affect protein and RNA levels (Figure 2H,I).

### 3.4 EVs and EPs Carry Distinct RNA Profiles, With EV‐Associated RNA Shorter Than 200 Nucleotides

**Figure 3:**
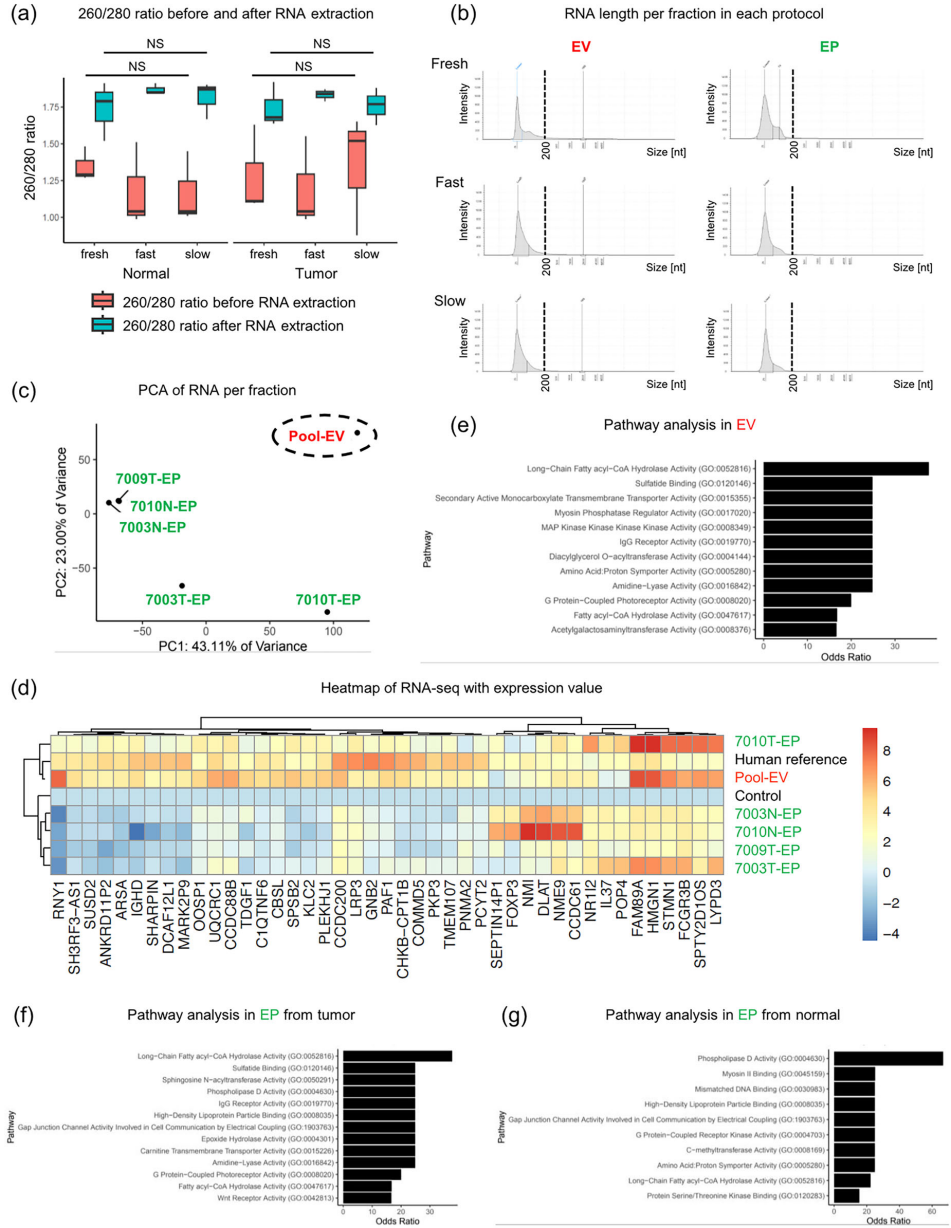
**Transcriptomic analysis**. (A) 260/280 absorbance ratio measured by NanoDrop before and after RNA extraction in each storage protocol. Values are indicated as medians (*n* = 10, per group, fresh, fast or slow). The significance of differences was calculated using the nonparametric Wilcoxon matched‐pairs signed‐rank test. “NS” indicates no statistically significant difference. (B) RNA size and intensity of EV and EP fraction (fractions 1–4 and 5–10) in each storage protocol were measured by TapeStation (7015 tumour). The 200 nt line is marked with a dotted line. (C) Principal components analysis (PCA) of RNA from five fresh lung samples. Due to the low concentrations of fractions 1–4 (EV), five samples (7003 tumour, 7003 normal, 7009 tumour, 7010 tumour, and 7010 normal) were pooled for analysis (Pool‐EV). This “Pool‐EV” is circled by dotted lines. (D) Top RNA signatures enriched in each sample. The human reference and control are included for comparative purposes. “Pool‐EV” represents the combined EV fractions from five samples. (E‐G) Pathway analysis showing the top biological processes enriched in EV and EP fraction (fraction 1–4 and 5–10) from tumour or normal tissue (E: fraction 1–4 (EV), F: fraction 5–10 (EP) [tumour], G: fraction 5–10 (EP) [normal]).

We examined RNA characteristics in EVs and EPs using lung (normal) and prostate (tumour) tissues. A total of 18 samples were prepared using three protocols: fresh, frozen‐fast, and frozen‐slow (Table 3). Each sample was fractionated by SEC into EV and EP fractions. As a quality control, RNA purity, measured by 260/280 absorbance ratios, increased significantly after extraction across all protocols, with no differences (*P* > 0.05) between fresh and frozen samples (Figure 3A).

**Table 3:** A list of samples for capillary electrophoresis.

Patient	Cancer type	Tissue type	Storage	Fraction	RNA before	260/280 before	RNA after	260/280 after
7015	prostate	tumour	fresh	EV	13.1	1.63	14.2	1.68
7015	prostate	tumour	fresh	EP	302.1	1.11	112.7	1.92
7015	prostate	tumour	frozen‐fast	EV	23.8	1.55	20.2	1.84
7015	prostate	tumour	frozen‐fast	EP	371.4	0.99	111.2	1.87
7015	prostate	tumour	frozen‐slow	EV	25.9	1.52	23.1	1.88
7015	prostate	tumour	frozen‐slow	EP	239	0.88	74.3	1.77
7010	lung	normal	fresh	EV	95	1.29	23.4	1.79
7010	lung	normal	fresh	EP	735.8	1.27	80.7	1.91
7010	lung	normal	frozen‐fast	EV	40.3	1.51	68	1.85
7010	lung	normal	frozen‐fast	EP	430.1	1.04	163.7	1.91
7010	lung	normal	frozen‐slow	EV	57.9	1.45	18.2	1.67
7010	lung	normal	frozen‐slow	EP	747.6	1.04	409.6	1.90

*Note*: EV refers to fractions 1–4 and EP to fractions 5–10. The values before and after RNA extraction are shown in ng/µL.

To further assess the RNA composition, we first used capillary electrophoresis and demonstrated that the RNA in EV and EP fractions was predominantly short, ranging from 25 to 200 nucleotides, and RNA concentrations and integrity were comparable across protocols (Figure 3B). As the classic RNA integrity number does not apply to shorter than 75 pb reads, we inspected the RNA profiles visualised as recommended (Miceli et al. 2024).

Next, we pooled EV (F1‐4) and other EP (F11‐) fractions obtained from fresh lung samples due to the lower concentration of RNA and protein in them (Table 4), and performed quality control (Figure S3A–D), followed by small RNA sequencing (RNA‐seq). The principal component analysis (PCA) showed distinct separation of EVs from EPs fractions, indicating a unique RNA profile per type of vesicle or particle (Figure 3C). Overall, these results support previous reports (Miceli et al. 2024) that EVs and EPs carry different types of RNA and have differential transcriptomic profiles.

**Table 4:** A list of samples for RNA sequences.

Sample	Tissue type	Fraction	Concentration (ng/uL)	RIN	Volume (uL)
7003	tumour	EV	9.92	1.6	6.0
7003	normal	EV			
7009	tumour	EV			
7010	tumour	EV			
7010	normal	EV			
7003	tumour	EP	1056	2.6	1.0
7003	normal	EP	184	2.6	1.1
7009	tumour	EP	85.6	2.6	2.3
7010	tumour	EP	75.4	2.6	2.7
7010	normal	EP	1136	N/A	1.0
7003	tumour	other EP	5.52	N/A	6.0
7003	normal	other EP			
7009	tumour	other EP			
7010	tumour	other EP			
7010	normal	other EP			

*Note*: EV refers to fractions 1–4, EP to fractions 5–10, and other EP to fractions from 11 onward.

Abbreviation: RIN, RNA Integrity Number.

Additionally, we investigated the gene ranking across different fractions and showed that the EV fraction (Pool‐EV) displayed a prominent expression of *RNY1*, a small non‐coding RNA from the Y RNA family. Consistent with previous reports (Miceli et al. 2024; Shurtleff et al. 2017), Y RNAs such as *RNY1* have been described as components of EVs, suggesting its potential involvement in intercellular communication or RNA quality control mechanisms mediated by these vesicles. Fragmented messenger RNAs (mRNAs) have been reported to be present in EVPs (Chiang et al. 2023; Miceli et al. 2024), and indeed, mRNAs such as *FAM89A*, *HMGN1*, *STMN1*, and *LYPD3* were detected in the EVPs. Notably, the EP fraction exhibited differential RNA expression between tumour and normal tissues, with genes such as *SEPTIN14P1*, *FOXP3*, *NMI*, *DLAT*, *NME9*, and *CCDC61* being more highly expressed in the normal tissues (Figure 3D and S3E), which play roles in diverse cellular processes, including signalling, nucleotide metabolism, and cell division. This suggests that the EVPs secreted from the tumour and normal tissues of cancer patients may have distinct RNA profiles. For instance, *STMN1* (a key regulator of microtubule dynamics often overexpressed in cancer) and *LYPD3* (implicated in cell adhesion and cancer progression) potentially influence recipient cell behaviour. Also, the higher expression of *FOXP3* (a master regulator of immune‐suppressing regulatory T cells) observed in normal tissues could indicate a suppressed or altered immune microenvironment in the nearby tumour.

Finally, pathway analysis using the R package EnrichR indicated a predominant upregulation of metabolic, cellular signalling, and immune‐related pathways in the EV fractions (Figure 3E). In the EP fraction, fatty acid metabolism pathways were observed in both tumour and normal tissues, whereas immune‐related pathways were upregulated only in tumour tissues. Additionally, pathways associated (*P* < 0.05) with gene and epigenomic regulation, such as mismatched DNA binding and C‐methyltransferase, were specifically observed in normal tissues (Figure 3F,G). Thus, in the EP fractions, distinct differences in associated pathways were observed between tumour and normal tissues.

### 3.5 EVP Proteomic Profiles Remain Stable Between Fresh and Frozen Samples

**Figure 4:**
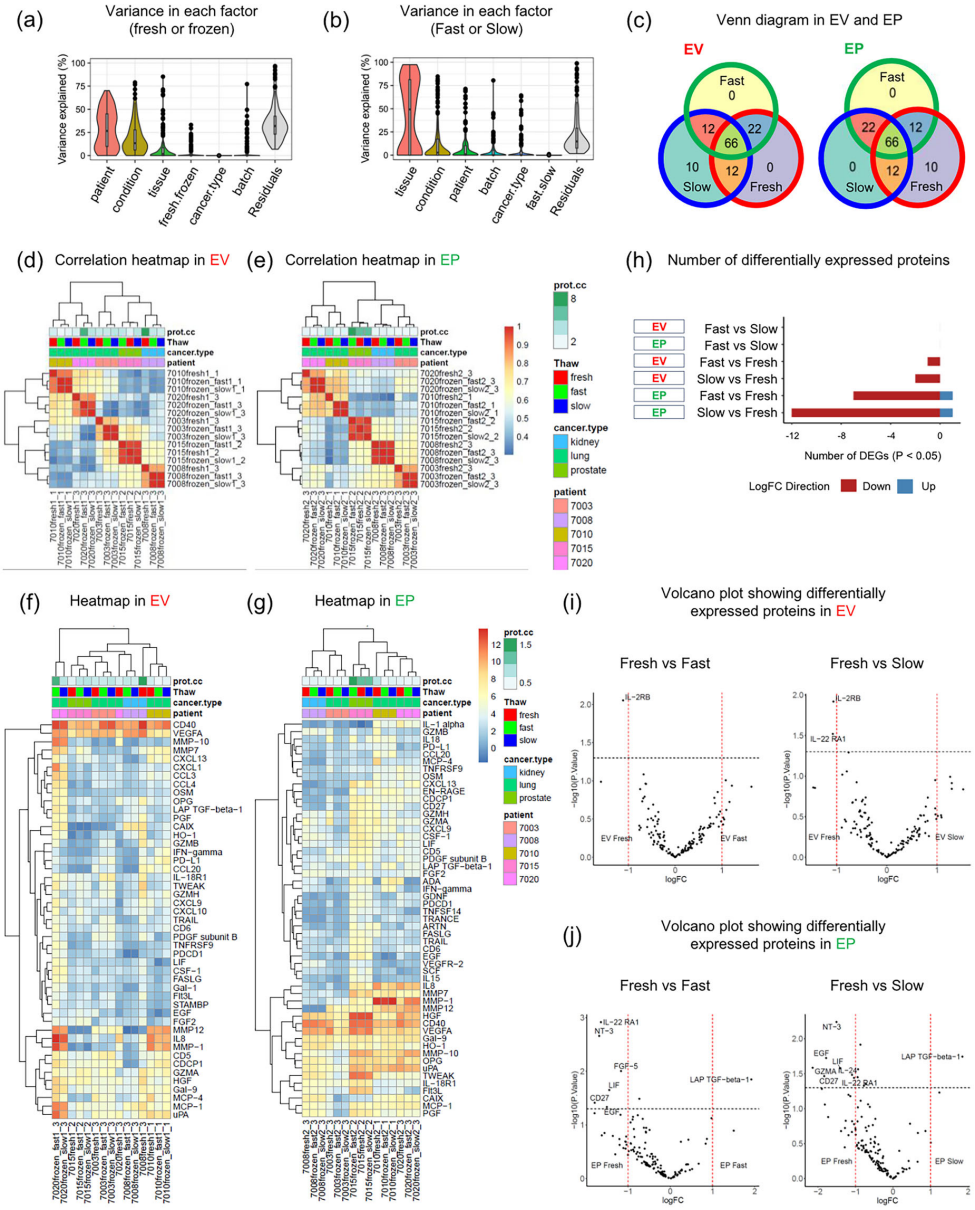
**Olink proteomics analysis of fresh and frozen samples for EVs and EPs**. (A–B) Percent variance explained associated with patient, condition, tissue, cancer type, batch, residuals, and storage protocol ((a) fresh or frozen; (b) Fast or Slow). (A) “fresh.frozen” represents the variance between fresh and frozen (*n* = 15, 3 samples per patient at unique thawing conditions). (B) “fast.slow” represents the variance between fast and slow. (C) Overlap of differentially expressed proteins between each storage protocol in EV and EP fractions. The top 100 proteins ranked per protocol are indicated. (D–E) Correlation heatmap of proteomics from tissue samples in EVs (D) and EPs (E). (F–G) Heatmap of protein expression profiles in fresh versus frozen EVs (F) and EPs (G) derived from tissue samples. (H) The number of differentially expressed proteins (*P* < 0.05) between each comparison among EV and EP fractions. No proteins with significant differences were detected after FDR correction. (I–J) Differentially expressed proteins (*P* < 0.05) in Fresh, Fast, and Slow protocols of fresh versus frozen in EVs (I) and EPs (J). Vertical dashed lines indicate fold change of 1.0; horizontal line indicates *P* value = 0.05. Red dots indicate proteins with fold changes > 1.5 or ←1.5, and *P* values < 0.05. Red dots indicate significantly differentially expressed proteins.

We next analysed tissue‐derived EVP samples to assess the impact of different preservation methods (Fresh, Fast, and Slow) on immune‐oncology related proteins in both the EV and EP fractions. Initially, we confirmed that overall variance between fresh and frozen samples was minimal (Figure 4A). In particular, differences in protein expression between Fast and Slow protocols were negligible (Figure 4B). These differentially expressed proteins showed substantial overlap across the three protocols in both EVs and EPs (Figure 4C). The correlation between EVs and EPs shows that fresh and frozen samples from the same patient were highly similar (Pearson and Spearman > 0.95 and *P* < 0.05, Figure 4D,E). Quantification of EVs revealed consistent detection of CD40 and VEGFA across all patient samples, while inflammatory proteins, including MMP12, IL‐8 and MMP‐1, exhibited notable inter‐patient variability and were more abundantly expressed in lung cancer‐derived samples (Figure 4F). A similar trend was seen in EPs, where IL8, MMP‐1, and other proteins showed considerable variability among patients, while protein expression profiles were largely comparable between fresh and frozen samples from the same individual (Figure 4G). In the PCA of tissue samples, there was a large overlap between EVs and EPs, similarly, differences based on cancer type were also minimal (Figure S4A,B).

When comparing Fresh, Fast, and Slow samples within both EVs and EPs, a few proteins were differentially expressed at nominal *P* < 0.05. However, no proteins remained statistically significant after false discovery rate (FDR) correction (Figure 4H and S4C). Proteins showing nominal *P*‐value differences are highlighted in volcano plots (Figure 4I,J). In EVs, IL‐2RB expression was higher in Fresh compared to Fast, and both IL‐2RB and IL‐22RA1 were elevated in Fresh compared to Slow (Figure 4I). In EPs, TGF‐β1 expression was lower in Fresh than in Fast, while IL‐22RA1, NT‐3, FGF‐5, LIF, CD27, and EGF were detected at higher levels in fresh samples. Similarly, when comparing Fresh to Slow EPs, the level of TGF‐β1 was again lower, and NT‐3, EGF, GZMA, LIF, IL‐24, CD27, and IL‐22RA1 were all elevated in fresh samples (Figure 4J). Notably, no proteins were significantly different between the Fast and Slow groups in either EVs or EPs (Figure S4D,E). Finally, EVP samples were analysed focusing on the differences between EVs and EPs. Interestingly, the majority of proteins were more highly expressed in EPs than in EVs. The only exception was CD4, which showed higher expression in EVs (Figure S4F–H).

### 3.6 EVP Proteomic Characteristics Show Heterogeneity Across Tissues

**Figure 5:**
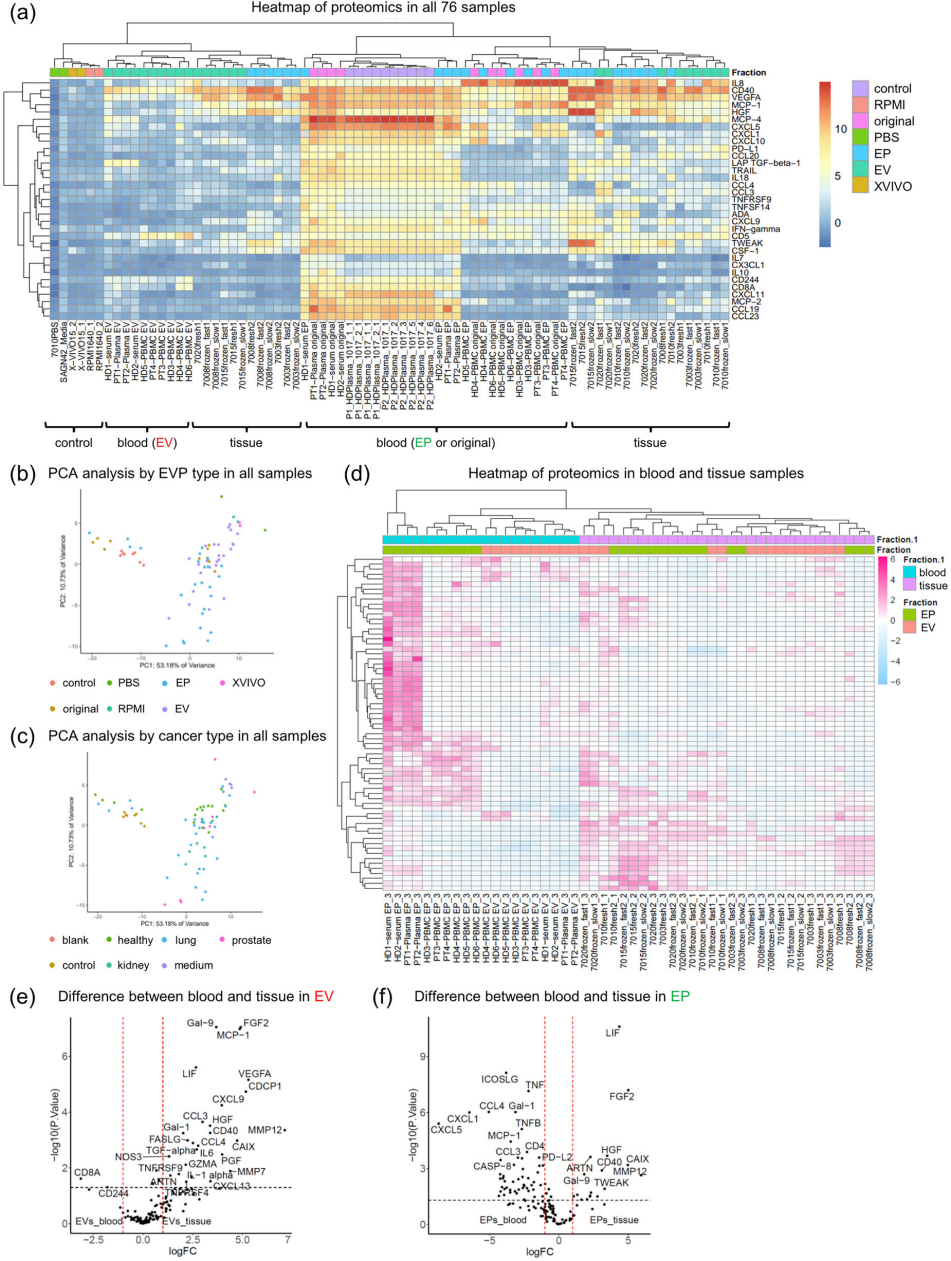
**Olink proteomics analysis of different types of tissue. (A)** Hierarchical clustering heatmap of all 76 samples (15 patients total, 9 cancer patiens and 6 normal controls), with EV, EP, original (pre‐size‐ exclusion chromatography) samples, and positive and negative controls colour‐coded accordingly. Control: human healthy donor serum (HD1017), medium: X‐VIVO, RPMI 1640, and PBS, Original: before size‐exclusion chromatography. The scale indicates protein expression in the log_2_ normalised scale. (B–C) Principal component analysis (PCA) of proteomics from all samples. (B: analysis by EVP type, C: cancer type) (D) Heatmap analysis of EVPs derived from blood and tissue. EVs and EPs are further colour‐coded separately. (E–F) Volcano plots showing the differentially expressed proteins (*P *< 0.05) between tissue and blood samples in EVs (E) and EPs (F). Vertical dashed lines indicate fold change of 1.0; horizontal line indicates *P* value = 0.05. Red dots indicate proteins with fold changes > 1.5 or ←1.5, and *P* values < 0.05. Red dots indicate significantly differentially expressed proteins.

To further investigate the differences in proteomic profiles of EVPs by tissue origin, we first compared the EVPs derived from different tissue samples and blank controls, such as media or unfiltered commercial buffers represented by PBS and RMPI (Figure 5A). In total, 76 samples, including 30 tissue‐supernatants, 12 serum or plasma, 18 PBMC‐culture medium, and 16 controls, were subjected to Olink and mixed effect modelling analysis (Table 5). As expected, EVP proteomes were clustered primarily according to sample origin. Blood‐derived EVPs from serum, plasma, and PBMCs were clearly stratified into EV and EP groups. The “original” sample before SEC from blood showed characteristics similar to EPs, which may reflect the low protein content in the EV fractions of blood samples. PCA analysis confirmed the separation between EVs and EPs, with most samples forming distinct clusters, although some overlap was observed (Figure 5B,C). Subsequent analysis of EV or EP samples from blood and tissue demonstrated clear clustering by origin, emphasising the pronounced influence of tissue source on the proteomic profile of EVPs (Figure 5D). These differences were further illustrated by comparing the proteomes of blood‐ and tissue‐derived EVPs (Figure 5E,F). CD8A was markedly enriched in blood‐derived EVs, whereas extracellularly active secreted proteins, including Gal‐9, FGF2, and MCP‐1, were predominantly found in tissue‐derived EVs. Conversely, in EPs, proteins such as TNF, Gal‐1, and CCL4 were enriched in blood, while LIF and FGF2 were more prominent in tissue‐derived EPs. Interestingly, tissue‐derived proteins were more prominently detected in EVs, while blood‐derived proteins were more strongly detected in EPs.

**Table 5:** A list of samples for Olink proteomics.

Sample	Patient	Tissue type	Cancer type	Storage	Fraction	EV or EP
7010fresh1	7010	tissue	lung	fresh	1‐4	EV
7010fresh2	7010	tissue	lung	fresh	5‐10	EP
7010frozen_fast1	7010	tissue	lung	frozen‐fast	1‐4	EV
7010frozen_fast2	7010	tissue	lung	frozen‐fast	5‐10	EP
7010frozen_slow1	7010	tissue	lung	frozen‐slow	1‐4	EV
7010frozen_slow2	7010	tissue	lung	frozen‐slow	5‐10	EP
7015fresh1	7015	tissue	prostate	fresh	1‐4	EV
7015fresh2	7015	tissue	prostate	fresh	5‐10	EP
7015frozen_fast1	7015	tissue	prostate	frozen‐fast	1‐4	EV
7015frozen_fast2	7015	tissue	prostate	frozen‐fast	5‐10	EP
7015frozen_slow1	7015	tissue	prostate	frozen‐slow	1‐4	EV
7015frozen_slow2	7015	tissue	prostate	frozen‐slow	5‐10	EP
7008fresh1	7008	tissue	kidney	fresh	1‐4	EV
7008fresh2	7008	tissue	kidney	fresh	5‐10	EP
7008frozen_fast1	7008	tissue	kidney	frozen‐fast	1‐4	EV
7008frozen_fast2	7008	tissue	kidney	frozen‐fast	5‐10	EP
7008frozen_slow1	7008	tissue	kidney	frozen‐slow	1‐4	EV
7008frozen_slow2	7008	tissue	kidney	frozen‐slow	5‐10	EP
7020fresh1	7020	tissue	lung	fresh	1‐4	EV
7020fresh2	7020	tissue	lung	fresh	5‐10	EP
7020frozen_fast1	7020	tissue	lung	frozen‐fast	1‐4	EV
7020frozen_fast2	7020	tissue	lung	frozen‐fast	5‐10	EP
7020frozen_slow1	7020	tissue	lung	frozen‐slow	1‐4	EV
7020frozen_slow2	7020	tissue	lung	frozen‐slow	5‐10	EP
7003fresh1	7003	tissue	lung	fresh	1‐4	EV
7003fresh2	7003	tissue	lung	fresh	5‐10	EP
7003frozen_fast1	7003	tissue	lung	frozen‐fast	1‐4	EV
7003frozen_fast2	7003	tissue	lung	frozen‐fast	5‐10	EP
7003frozen_slow1	7003	tissue	lung	frozen‐slow	1‐4	EV
7003frozen_slow2	7003	tissue	lung	frozen‐slow	5‐10	EP
PT1‐Plasma original	PT1	blood	lung	N/A	before SEC	original
PT1‐Plasma EV	PT1	blood	lung	N/A	1‐4	EV
PT1‐Plasma EP	PT1	blood	lung	N/A	5‐10	EP
PT2‐Plasma original	PT2	blood	lung	N/A	before SEC	original
PT2‐Plasma EV	PT2	blood	lung	N/A	1‐4	EV
PT2‐Plasma EP	PT2	blood	lung	N/A	5‐10	EP
HD1‐serum original	HD1	blood	healthy	N/A	before SEC	original
HD1‐serum EV	HD1	blood	healthy	N/A	1‐4	EV
HD1‐serum EP	HD1	blood	healthy	N/A	5‐10	EP
HD2‐serum original	HD2	blood	healthy	N/A	before SEC	original
HD2‐serum EV	HD2	blood	healthy	N/A	1‐4	EV
HD2‐serum EP	HD2	blood	healthy	N/A	5‐10	EP
PT3‐PBMC original	PT3	blood	lung	N/A	before SEC	original
PT3‐PBMC EV	PT3	blood	lung	N/A	1‐4	EV
PT3‐PBMC EP	PT3	blood	lung	N/A	5‐10	EP
PT4‐PBMC original	PT4	blood	lung	N/A	before SEC	original
PT4‐PBMC EV	PT4	blood	lung	N/A	1‐4	EV
PT4‐PBMC EP	PT4	blood	lung	N/A	5‐10	EP
HD3‐PBMC original	HD3	blood	healthy	N/A	before SEC	original
HD3‐PBMC EV	HD3	blood	healthy	N/A	1‐4	EV
HD3‐PBMC EP	HD3	blood	healthy	N/A	5‐10	EP
HD4‐PBMC original	HD4	blood	healthy	N/A	before SEC	original
HD4‐PBMC EV	HD4	blood	healthy	N/A	1‐4	EV
HD4‐PBMC EP	HD4	blood	healthy	N/A	5‐10	EP
HD5‐PBMC original	HD5	blood	healthy	N/A	before SEC	original
HD5‐PBMC EV	HD5	blood	healthy	N/A	1‐4	EV
HD5‐PBMC EP	HD5	blood	healthy	N/A	5‐10	EP
HD6‐PBMC original	HD6	blood	healthy	N/A	before SEC	original
HD6‐PBMC EV	HD6	blood	healthy	N/A	1‐4	EV
HD6‐PBMC EP	HD6	blood	healthy	N/A	5‐10	EP
P1_HDPlasma_1017_1	control	blood	control	control	control	control
P1_HDPlasma_1017_2	control	blood	control	control	control	control
P2_HDPlasma_1017_1	control	blood	control	control	control	control
P2_HDPlasma_1017_2	control	blood	control	control	control	control
P1_HDPlasma_1017_1	control	blood	control	control	control	control
P1_HDPlasma_1017_2	control	blood	control	control	control	control
P2_HDPlasma_1017_3	control	blood	control	control	control	control
P2_HDPlasma_1017_4	control	blood	control	control	control	control
P2_HDPlasma_1017_5	control	blood	control	control	control	control
P2_HDPlasma_1017_6	control	blood	control	control	control	control
7010PBS_1	blank	control	blank	blank	PBS	PBS
SAGN42_Media_1	blank	control	blank	blank	PBS	PBS
X‐VIVO15_1	medium	control	medium	medium	1‐4	XVIVO
X‐VIVO15_2	medium	control	medium	medium	5‐10	XVIVO
RPMI1640_1	medium	control	medium	medium	1‐4	RPMI
RPMI1640_2	medium	control	medium	medium	5‐10	RPMI

Abbreviations: EVs, extracellular vesicles; EPs, extracellular particles; PBMC, peripheral blood mononuclear cell; RPMI, Roswell Park Memorial Institute (RPMI) 1640; SEC, size‐exclusion chromatography.

## 4 Discussion

In this study, we assessed the impact of long‐term freezing on tissue‐derived EVP analyses. Tumour and matched normal tissues stored between 5 months up to a little over 2 years (172‐731 days), prior to EVP isolation showed no significant loss in RNA or protein yield, and key particle characteristics were preserved. Size distribution, as determined by DLS and Western blot, confirmed that EVs remained enriched in early SEC fractions (F1‐4), while EPs predominated in later fractions (F5‐10), consistent with profiles obtained from fresh tissue. Small EPs are particles less than 50 nm in size and do not express canonical EV markers such as CD9 or TSG101. This population is considered to comprise exomeres, supermeres, and lipoproteins. These entities represent extracellular nanoparticles that lack a lipid bilayer membrane, yet encapsulate small RNAs and proteins (Miceli et al. 2024). Their biological functions remain largely unknown, and further detailed investigations are required. Importantly, freezing did not markedly alter EVP size profiles or RNA purity, and EV‐enriched fractions retained distinct RNA signatures relative to other fractions. Furthermore, Olink‐based proteomic profiling demonstrated strong concordance between fresh and frozen samples, indicating that overall proteomic signatures are maintained despite storage. Collectively, these findings support the feasibility of using frozen tissues for EVP isolation and multi‐omic characterization. This is particularly relevant for translational studies, as it expands the utility of archived biobank samples and enables retrospective analyses without compromising data quality.

Several studies have examined the effects of fresh versus frozen storage conditions on the concentration, particle size, surface marker expression, protein profiles, and RNA content of EVPs derived from biofluids and cell culture media (Ahmadian et al. 2024; Chung et al. 2021; Gelibter et al. 2022; Görgens et al. 2022; Kim et al. 2022; Kim et al. 2025; Klymiuk et al. 2024; Maroto et al. 2017; Muller et al. 2014; Sarker et al. 2014; Tsamchoe et al. 2023; Yuana et al. 2015). However, this is the first study to investigate tissue‐derived EVPs. Before summarising these findings, two important considerations should be noted. First, some studies isolate EVPs before freezing the samples, whereas others freeze the unprocessed samples and extract EVPs after thawing. Second, the duration of frozen storage varies among studies, which may influence the outcomes. Taking these factors into account, some reports have shown that freezing after EVP isolation leads to reduced particle numbers, increased particle size, and morphological changes (Gelibter et al. 2022; Görgens et al. 2022; Kim et al. 2025; Klymiuk et al. 2024; Maroto et al. 2017). In contrast, other studies report no significant alterations in these characteristics when samples are frozen prior to EVP extraction (Gelibter et al. 2022; Sarker et al. 2014; Yuana et al. 2015). In our study, tissue slurries were frozen before EVP isolation, which may have minimised the impact of freezing on particle properties. There are relatively few reports investigating the downstream proteomic and transcriptomic consequences of freezing. Moreover, proteomic analyses are typically performed using mass spectrometry, and several studies suggest that freezing has minimal impact on proteomic profiles in this context (Chung et al. 2021; Sarker et al. 2014; Tsamchoe et al. 2023). As for RNA analysis, although some studies have assessed yield and stability following freezing, with results ranging from no significant effect to a moderate reduction in RNA recovery (Chung et al. 2021; Görgens et al. 2022; Sarker et al. 2014), comprehensive analyses such as RNA‐seq profiling remain scarce (Kim et al. 2022).

Regarding protein composition, only one prior study has evaluated the impact of fresh versus frozen storage on tissue‐derived EVPs, reporting no significant differences in bulk characteristics such as protein concentration, particle size, or surface marker expression in hepatocellular carcinoma samples (Yang et al. 2024). However, that analysis did not include high‐resolution proteomic or transcriptomic profiling, leaving unresolved whether cryopreservation alters EVP molecular cargo.

To address this gap, we fractionated EVPs into EV‐ and EP‐enriched populations and performed targeted proteomic profiling using the Olink proximity extension assay, a sensitive and reproducible platform optimised for low‐input samples. Across fractions, protein abundance profiles were largely conserved between fresh and frozen conditions, indicating that cryostorage does not induce major proteomic remodelling of tissue‐derived EVPs. These findings extend prior bulk‐level observations by demonstrating molecular stability of EVP‐associated proteins following freezing. Collectively, our results suggest that cryopreservation predominantly affects recovery efficiency rather than qualitative cargo composition. Although tumour‐specific biology and methodological variability may contribute to subtle differences across studies, the overall stability observed here supports the use of frozen tissue as a reliable source for EVP proteomic analyses.

Our data provide molecular‐level evidence that EVPs isolated from cryopreserved tissues retain stable proteomic signatures when processed using standardised workflows. This expands the feasibility of leveraging archived specimens for EVP research while highlighting the importance of future studies across diverse tumour types and larger cohorts to define context‐dependent effects.

Although several studies have utilised the Olink assay for EVP proteomics (Ali Moussa et al. 2024; Bryl‐Górecka et al. 2018; Cano et al. 2023; Chandran et al. 2019; Coukos et al. 2008; Dorayappan et al. 2019; Ellegaard Nielsen et al. 2020; Gidlöf et al. 2019; Hyland et al. 2020; Kraaijvanger et al. 2025; Larssen et al. 2017; Maaninka et al. 2023; Manouchehri Doulabi et al. 2022; Norman et al. 2025; Pulliam et al. 2024; Sjoqvist and Otake 2022, 2023; Sjoqvist et al. 2020; Steiner et al. 2025; Sun et al. 2019; Tzaridis et al. 2023; Verwer et al. 2023; Viktorsson et al. 2022), all have focused on EVPs derived from biofluids or cell culture media, such as plasma, serum, urine, saliva, and ascites. To our knowledge, no prior studies have applied the Olink platform to tissue‐derived EVPs. Here, we performed Olink assays on EVPs isolated from tumour tissues, PBMCs, and serum, and demonstrated that EVP proteomes remain largely unaffected by tissue storage conditions, indicating that frozen tissue can be a reliable source for EVP‐based biomarker studies. In most Olink‐based EVP studies, proteolytic treatment using lysis buffer is commonly applied immediately before analysis to release internal proteins (Bryl‐Górecka et al. 2018; Cano et al. 2023; Chandran et al. 2019; Dorayappan et al. 2019; Ellegaard Nielsen et al. 2020; Gidlöf et al. 2019; Hyland et al. 2020; Larssen et al. 2017; Norman et al. 2025; Pulliam et al. 2024; Sjoqvist and Otake 2022, 2023; Sjoqvist et al. 2020; Steiner et al. 2025; Sun et al. 2019; Tzaridis et al. 2023; Verwer et al. 2023; Viktorsson et al. 2022). However, because our focus was on surface‐expressed proteins, we employed a non‐destructive protocol without lysis. This approach enabled the selective profiling of surface markers on intact EVPs, providing insights into their extracellular proteomic landscape. These findings have potential implications for the development of surface‐based EVP biomarkers. A limitation of our approach, however, is that it may have excluded the detection of intracellular or endogenous EVP proteins, which would require lysis‐based processing for comprehensive analysis. Future studies could include both approaches.

While this study shows that quantitatively that RNA and protein levels remain similar with between fresh and frozen samples, it also has several limitations. First, the relatively small sample size, inter patient variability and the limited types of cancer did not allow for deeper biological insights, especially for comparing the differences between the diverse RNAs discrepant between fresh and frozen samples, but it was appropriate as a technical comparison between storage conditions. Future studies should validate these findings using larger sample sizes across multiple cancer types. Second, heterogeneity is also expected to exist within each of the EV and EP fractions defined in this study. Third, following isolation, EVP fractions are typically frozen and stored for several months before downstream proteo‐transcriptomic analysis. Future studies could assess any impact this freezing step might have on the molecular characteristics of EVPs. Moreover, investigating the effects of long‐term freezing beyond one year will require the accumulation of additional samples in future studies. Fourth, the Olink proteomic analysis was restricted to two panels, Inflammation and Immuno‐Oncology, thereby covering only a subset of potentially relevant proteins. Furthermore, it should be acknowledged that the present study primarily profiled proteins expressed on the EVP surface and did not allow for conclusions regarding their relative abundance. Fifth, transcriptomic profiling of tissue‐derived EVPs is generally harder than profiling EVPs from biofluids, mainly because of extra contamination, lower/variable RNA yield, and the harsher sample processing required for tissues. Regarding the comparison between fresh and frozen samples, our evaluation was limited to proteomics, as RNA‐seq data were not available. In addition, this study does not examine the functional properties of tissue‐derived EVPs, and their physiological roles should be explored in future investigations. Finally, the cellular origin of the tissue‐derived EVPs remains unclear; the analysed vesicles could have originated from various cell types, including cancer cells, immune cells, fibroblasts, or other stromal components. More in‐depth molecular analyses, including integration of single‐cell RNA‐seq data, could help identify cell‐type‐specific EVPs.

## 5 Conclusion

In conclusion, this study demonstrated that while there are minor differences, protein profiles related to inflammation, immune response, and tumour immunology remained large unchanged between fresh and frozen EVPs derived from the same tissue samples. These findings suggest that frozen tissue may be a viable source for EVP‐based analyses, potentially increasing the utility of archived samples. Future work will aim to extend these findings to more tumour types and larger cohorts, and further investigate the biological relevance of tissue‐derived EVPs.

## Author Contributions

**Yohei Nose**: formal analysis, investigation, validation, data curation, methodology, writing – original draft, writing – review and editing. **Diane Marie del Valle**: resources, project administration, writing – review and editing. **Tina Ruth Gonsalves**: project administration, resources, writing – review and editing. **Kevin Tuballes**: investigation, methodology, writing – review and editing. **Ethan Ellis**: methodology, investigation. **Hui Xie**: methodology, investigation. **Igor Figueiredo**: methodology, software, formal analysis, visualisation. **Ruiwei Guo**: methodology, visualisation, software, formal analysis. **Avni Chandra**: investigation, formal analysis. **Aana Hahn**: methodology, visualisation. **Anish Korrapati**: methodology, investigation, visualisation. **Giorgio Ioannou**: methodology, investigation, visualisation. **Rafael Cabal**: methodology, investigation, visualisation. **Swapnil Tichkule**: methodology, investigation, visualisation. **John F. Fullard**: resources, methodology, visualisation, investigation. **Panos Roussos**: methodology, visualisation, investigation, resources. **Pedro Silva**: methodology, resources. **Angelo Amabile**: methodology, resources. **Jarod Morgenroth‐Rebin**: methodology, investigation. **Travis Dawson**: methodology, investigation. **Raphael Merand**: methodology, investigation. **Kai Nie**: methodology, investigation. **Zhihong Chen**: methodology, investigation. **Sharon Nirenberg**: methodology, investigation. **Brian Brown**: methodology, investigation. **Seunghee Kim‐Schulze**: methodology, investigation. **Andrew Kaufman**: methodology, investigation. **Raja Flores**: methodology, investigation. **Laura Zuluaga**: methodology, investigation. **Kristin Beaumont**: methodology, investigation. **Robert Sebra**: methodology, investigation. **Natasha Kyprianou**: methodology, investigation. **Kyrollis Attalla**: methodology, investigation, data curation. **Ketan Badani**: methodology, investigation, data curation. **Ash Tewari**: methodology, investigation. **Navneet Dogra**: methodology, investigation, writing – original draft, writing – review and editing, funding acquisition, supervision. **Sacha Gnjatic**: methodology, investigation, conceptualization, funding acquisition, validation, software, data curation, supervision, resources, formal analysis, project administration, writing – review and editing, visualisation, writing – original draft. **Edgar Gonzalez‐Kozlova**: methodology, investigation, conceptualisation, funding acquisition, data curation, supervision, resources, formal analysis, software, project administration, validation, writing – original draft, writing – review and editing, visualisation.

## Conflicts of Interest

The authors declare no competing interests. G.S. reports other research funding from Boehringer Ingelheim, Bristol‐Myers Squibb, Celgene, Genentech, Regeneron, and Takeda, not related to this paper.

## Supporting information



Supporting Information: jex270128‐sup‐0001‐SuppMat.xlsx

Supporting Information: jex270128‐sup‐0002‐FigureS1.jpg

Supporting Information: jex270128‐sup‐0003‐FigureS2.jpg

Supporting Information: jex270128‐sup‐0004‐FigureS3.jpg

Supporting Information: jex270128‐sup‐0005‐FigureS4.jpg

## Data Availability

The study did not generate new unique reagents. All data reported in the manuscript is available on Zenodo under ID:10.5281/zenodo.18499931 (https://zenodo.org/records/18499932) and upon request. All analysis code is available on GitHub (https://github.com/eegk/FreshFrozenEVPs) and upon request. This paper does not report any new code. Requests for further information, resources, and reagents should be directed to and will be fulfilled by the lead contact, Edgar Gonzalez‐Kozlova (edgar.gonzalez‐kozlova@mssm.edu).
