# Supplementary figures and images for: The Proteo‐Transcriptome of Extracellular Vesicles and Particles Is Largely Preserved After Cryopreservation
Source: J Extracell Biol. 2026 May 5;5(5):e70128. doi: 10.1002/jex2.70128 (PMC13140971; doi:10.1002/jex2.70128)

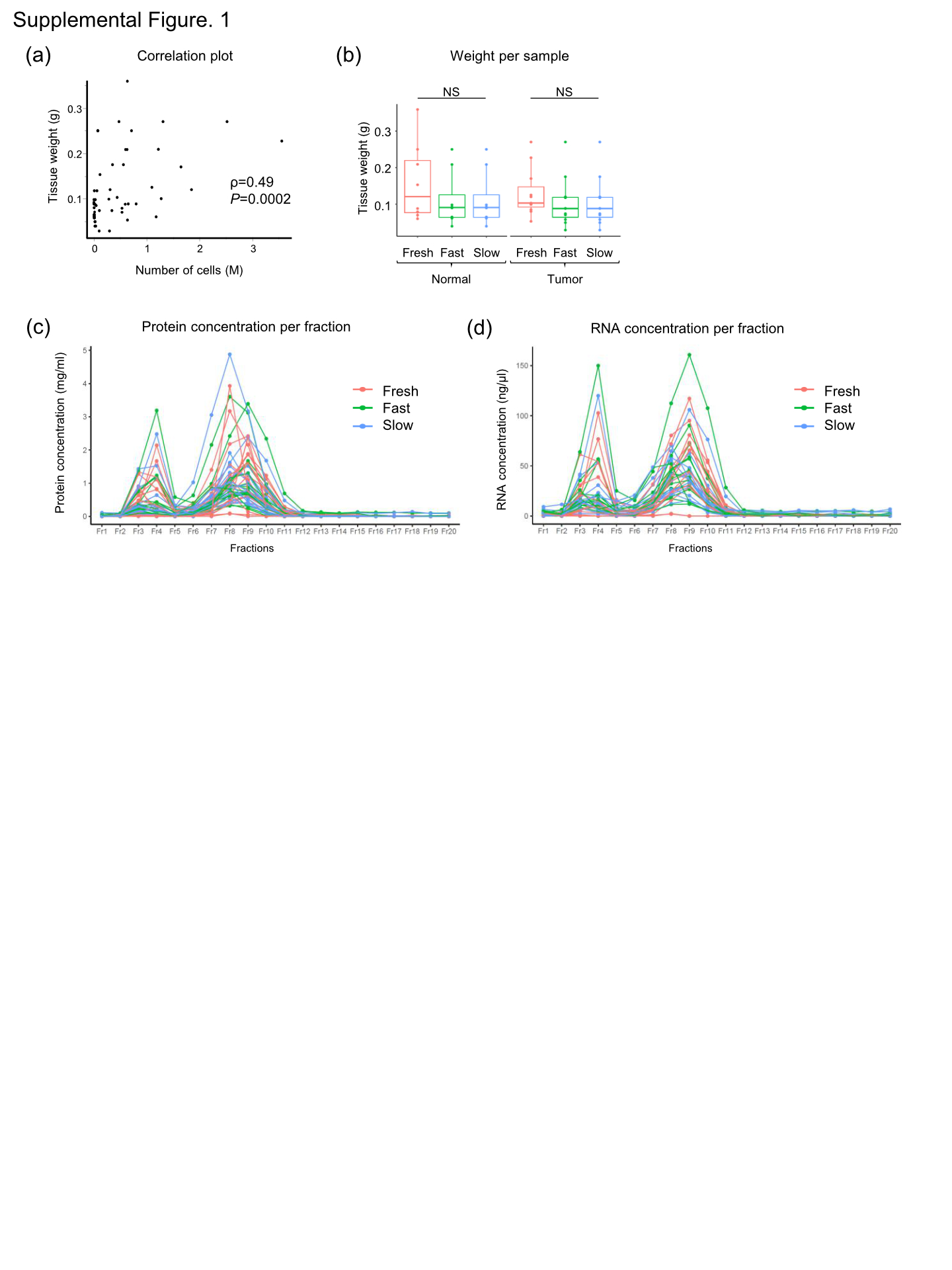

Supplement: Supplementary file 2 — Supporting Information: jex270128‐sup‐0002‐FigureS1.jpg [file JEX2-5-e70128-s004.jpg]

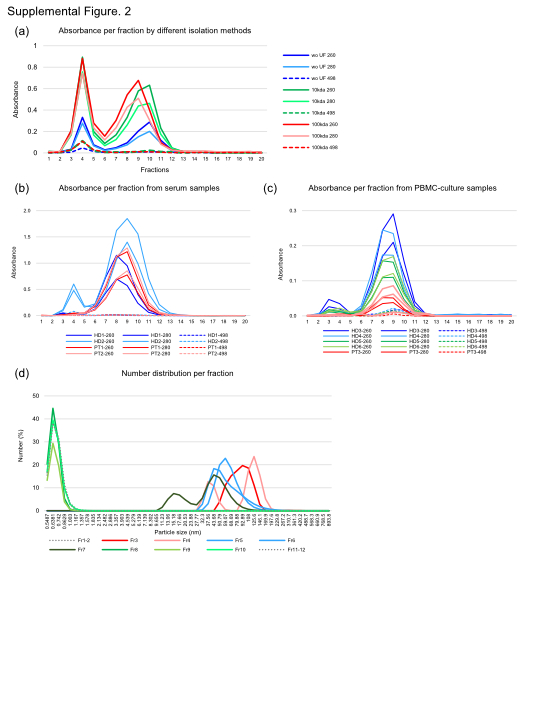

Supplement: Supplementary file 3 — Supporting Information: jex270128‐sup‐0003‐FigureS2.jpg [file JEX2-5-e70128-s003.jpg]

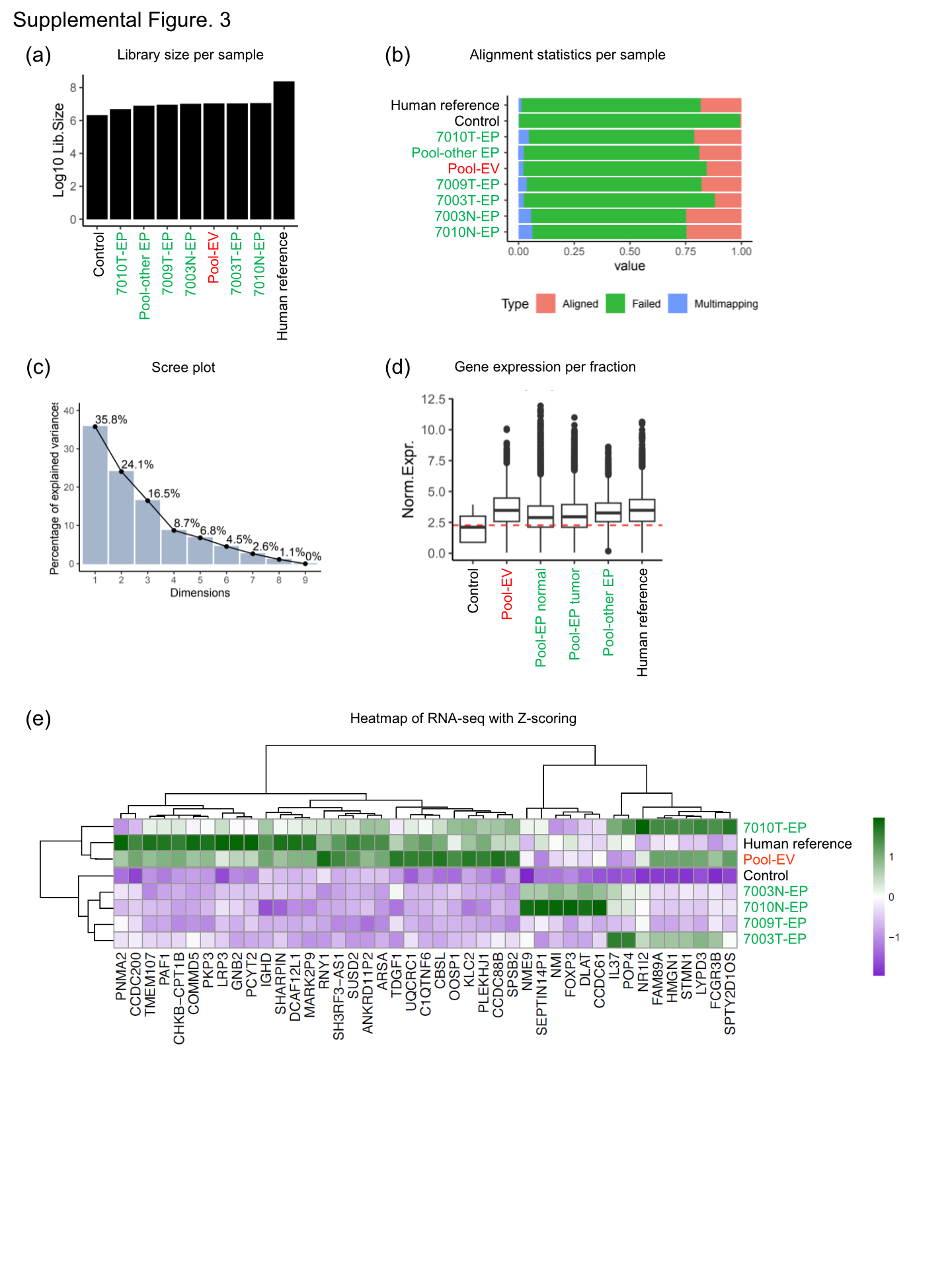

Supplement: Supplementary file 4 — Supporting Information: jex270128‐sup‐0004‐FigureS3.jpg [file JEX2-5-e70128-s005.jpg]

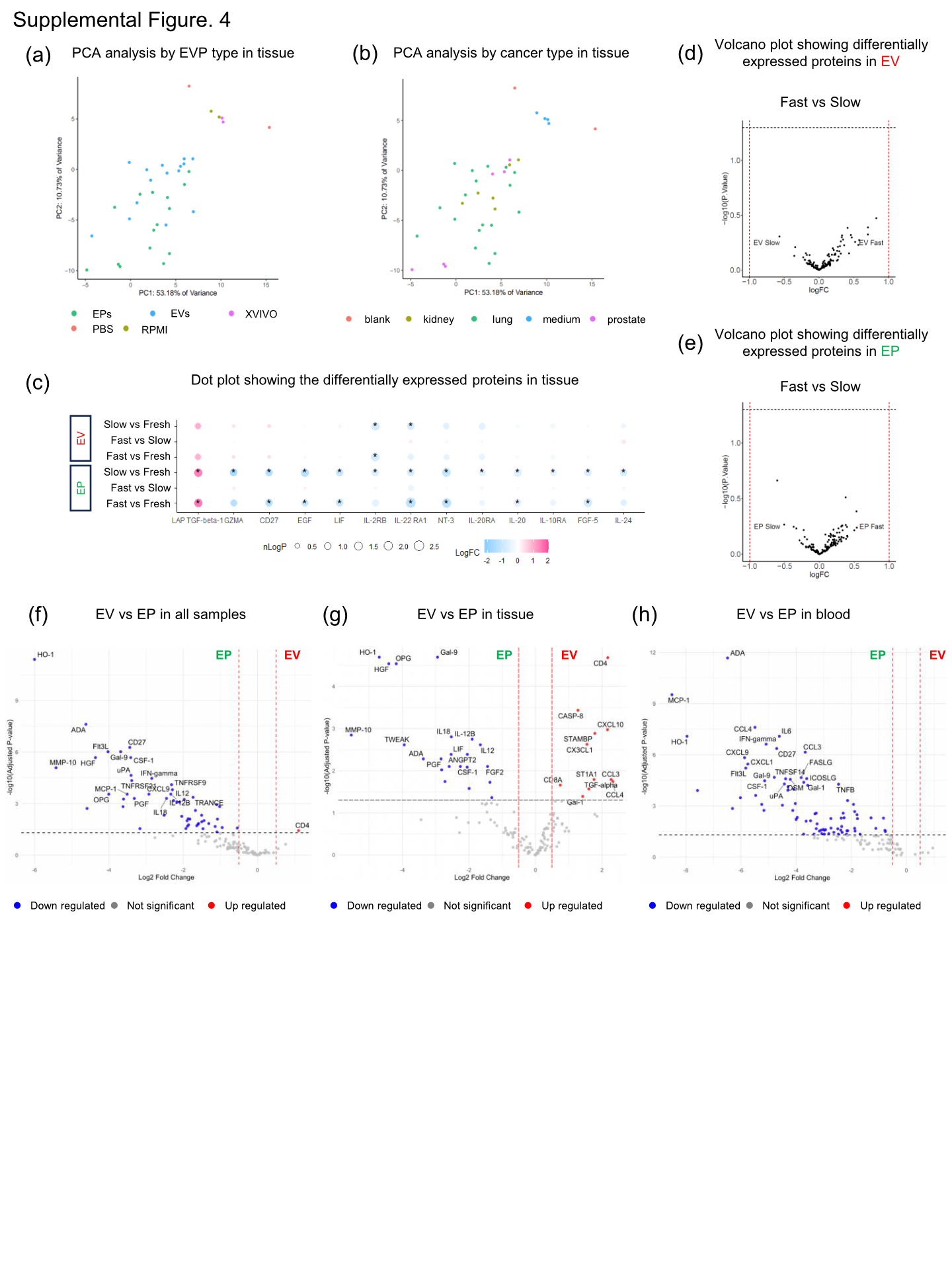

Supplement: Supplementary file 5 — Supporting Information: jex270128‐sup‐0005‐FigureS4.jpg [file JEX2-5-e70128-s001.jpg]
